# Regulations of the Apothecaries' Company; with Remarks on the Apprenticeship System

**Published:** 1830-01-01

**Authors:** 


### XIII.

### Regulations of the Apothecaries' Company.

"What," we think we hear our readers ejaculate in dismay, "A new curriculum from Apothecaries' Hall?" No, gentle student, not so bad as that; this is merely a resumé of the more than Herculean labours you must encounter before you face the dread examiners, a programme of the ceremonies for 1829 published by the Petronius to the "Company," Mr. Watson. They are in substance the regulations which we copied into the 19th number of this Journal, page 189 et seq. but they are licked into shape and clearness for the benefit of those now commencing their professional career.

"*Regulations to be observed by Students whose attendance on Lectures commenced before January 1st*, 1829.

"The Court of Examiners chosen and appointed by the Master, Wardens, and Assistants of the Society of Apothecaries, of the city of London, in pursuance of a certain act of parliament, 'for better regulating the practice of Apothecaries throughout England and Wales,' passed in the fifty-fifth year of the reign of his Majesty King George the Third, apprise all persons whom it may concern:

"That every candidate for a certificate to practise as an apothecary, will be required to possess a competent knowledge of the Latin language, and, in compliance with the fourteenth and fifteenth sections of the said act, to produce testimonials of having served an apprenticeship of not less than five years to an apothecary, of having attained the full age of twenty-one years, and of good moral conduct.

"Candidates will also be required to produce testimonials of attendance on lectures and medical practice agreeably to regulations at different times published by the Court.

"Those whose attendance on lectures commenced prior to the 1st of February, 1828, will be admitted to examination after an attendance on one course of lectures on Chemistry, one course of lectures on Materia Medica, two courses of lectures on Anatomy and Physiology, two courses of lectures on the Theory and Practice of Medicine, and six months physician's practice at an hospital, or nine months at a dispensary.

"Those who began to attend lectures subsequently to the 1st of February, 1828, and previously to the 1st of October in the same year, will only be admitted to examination after the following course of study—viz. an attendance on one course of lectures on Chemistry, one course of lectures on Materia Medica and Botany, two courses of lectures on Anatomy and Physiology, two courses of lectures on the Theory and Practice of Medicine—to be attended subsequently to the lectures on Chemistry and Materia Medica, and to one course at least of Anatomy—and six months, at least, physician's practice at an hospital, or nine months at a dispensary; such attendance to commence subsequently to the termination of the first course of lectures on the Principles and Practice of Medicine.

"Those whose attendance on lectures commenced on or after the 1st of October, 1828, and previously to the 1st of January, 1829, will be required to produce testimonials of having attended two courses of lectures on Chemistry, two courses of lectures on Materia Medica and Botany, two courses of lectures on Anatomy and Physiology, two courses of Anatomical Demonstrations, two courses of lectures on the Theory and Practice of Medicine—to be attended subsequently to one course of lectures on Chemistry, Materia Medica, and Anatomy—and six months, at least, the physicians' practice at an hospital (containing not less than sixty beds), or nine months at a dispensary: such attendance to commence subsequently to the termination of the first course of lectures on the Principles and Practice of Medicine.

"*Regulations to be observed by Students whose attendance on Lectures commenced since January 1st*, 1829.

"The Court of Examiners chosen and appointed by the Master, Wardens, and Assistants of the Society of Apothecaries, of the city of London, in pursuance of a certain act of Parliament, 'for better regulating the practice of Apothecaries throughout England and Wales,' passed in the fifty-fifth year of the reign of his Majesty King George the Third, apprise all persons whom it may concern:

"That every candidate for a certificate to practise as an apothecary, will be required to possess a competent knowledge of the Latin language, and, in compliance with the fourteenth and fifteenth sections of the said act, to produce testimonials of having served an apprenticeship of not less than five years to an apothecary, of having attained the full age of twenty-one years, and of good moral conduct; and also testimonials of having attended two courses of lectures on Chemistry; two courses of lectures on Materia Medica, Therapeutics, and Botany; two courses of lectures on Anatomy and Physiology; two courses of Anatomical Demonstrations; two courses of lectures on the Theory and Practice of Medicine—to be attended subsequently to one course of lectures on Chemistry, Materia Medica, and Anatomy; two courses of lectures on Midwifery and the Diseases of Women and Children; and nine months, at least, the physician's practice at an hospital (containing not less than sixty beds), or twelve months at a dispensary: such attendance to commence subsequently to the termination of the first course of lectures on the Principles and Practice of Medicine.

" Students are, moreover, earnestly recommended to attend Clinical Lectures, and diligently to avail themselves of instruction in Morbid Anatomy and Forensic Medicine.

" The examination of the candidate will be as follows:

" 1. In translating grammatically parts of the Pharmacopœia Londinensis, and physicians' prescriptions; and after the 1st of January, 1831, candidates will be required to translate portions of the following medical Latin authors—viz. Celsus de Medicinâ, or Gregory Conspectus Medicinæ Theoreticæ.

" 2. In Chemistry.

" 3. In Materia Medica and Therapeutics.

" 4. In Botany.

" 5. In Anatomy and Physiology.

" 6. In the Practice of Medicine.

" N.B.—Physicians' pupils, who intend to present themselves for examination, must appear personally at the Beadle's office, in this Hall, and bring with them the tickets, authorizing their attendance on such practice, as the commencement thereof will be dated from the time of such personal appearance.

" No testimonial of attendance on lectures on the Principles and Practice of Medicine, delivered in London, or within seven miles thereof, will render a candidate eligible for examination, unless such lectures were given, and the testimonial is signed by, a fellow, candidate, or licentiate, of the Royal College of Physicians of London.

" *Notice.*—Every person intending to qualify himself under these regulations, to practise as an apothecary, may obtain, at the Beadle's office, at this Hall, (where attendance is given every day, except Sunday, from nine until two o'clock), a printed form of certificate of all the lectures candidates are required to attend, and also of the physician's practice. The Court requests the blanks may be filled up when signed by the respective Lecturers and Physicians whose lectures or practice the student has attended.

" Students are enjoined to observe, that, in future, these certificates, so filled up, will be required from candidates for examination, and that no other form of testimonials of attendance on lectures and medical practice will be admitted, except such certificates as have heretofore been received, if the same were obtained prior to the 1st of February, 1828; or such as bear the seal of a University or College, and the signature of the officer attached to such University or College whose duty it is to sign certificates of attendance on the lectures given therein.

" Every person offering himself for examination must give notice in writing, to the Clerk of the Society, on or before the Monday previously to the day of examination; and must also, at the same time, deposit all the required testimonials at the office of the Beadle.

" The Court will meet in the Hall every Thursday, where candidates are required to attend at half-past four o'clock.

" By order of the Court,

" JOHN WATSON, Sec.

" London, Sept. 1, 1829.

"It is expressly ordered by the Court of Examiners, that no gratuity be received by any officer from any person applying for information relative to the business of this Court."

Having before expressed our opinion on the Curriculum, few remarks are required on the present occasion. We must once more protest against the studied ambiguity with which the apprenticeship is mentioned. It is obviously left to the discretion and generosity of the master to grant or deny permission to the apprentice, to dedicate part of the long and unprofitable "five years" to attendance on the prescribed courses of lectures. Can any thing be more iniquitous than placing an unfortunate young man behind a counter for five whole years, engaged in the wholesome and intellectual employment of rolling pills and papering bottles? As the regulations stand, there is no prohibition certainly against a portion of this time being occupied in hospital and other attendance, but the mischief of it is, that such a course is not *enjoined*, is not rendered *imperative* on master and man. In London the apprentices of the respectable general practitioners have no reason to complain, for we *know*, and we speak it to their honour, that the latter afford them every possible facility and advantage in the prosecution of their studies. But laws are for the bad: the good can do without them. The great mass of apprentices in the country and many in this town, are bound by the letter and to the term of their indenture—are Serfs and Helots as long as their blindly interested masters can compel them by the Gordian shackles of the law to be so! No doubt the directors of the Apothecaries' Company have formidable prejudices and interests to contend with in any attempt at altering the apprenticeship system; but let them do what is right, and the support of the well informed members of their profession will bear them triumphantly through a more desperate struggle.

We cannot conclude without expressing our general approbation of the spirit and details of the present regulations. The Apothecaries' Company have raised themselves in public estimation by the desire they have shewn to enhance the qualifications, and of course the character, of those who are about to become members of their body. The young men who *now* pass the Hall, may step into society with the proud consciousness that their acquirements are of a stamp, far, far above that of the Apothecaries of twenty years ago.

